# SARS-CoV-2 Antibody Prevalence among Industrial Livestock Operation Workers and Nearby Community Residents, North Carolina, 2021 to 2022

**DOI:** 10.1128/msphere.00522-22

**Published:** 2023-01-19

**Authors:** Carolyn Gigot, Nora Pisanic, Kate Kruczynski, Magdielis Gregory Rivera, Kristoffer Spicer, Kathleen M. Kurowski, Pranay Randad, Kirsten Koehler, William A. Clarke, Phyla Holmes, D. J. Hall, Devon J. Hall, Christopher D. Heaney

**Affiliations:** a Department of Environmental Health and Engineering, Johns Hopkins Bloomberg School of Public Health, Baltimore, Maryland, USA; b Department of Pathology, Johns Hopkins University School of Medicine, Baltimore, Maryland, USA; c Rural Empowerment Association for Community Help, Warsaw, North Carolina, USA; d Department of Epidemiology, Johns Hopkins Bloomberg School of Public Health, Baltimore, Maryland, USA; e Department of International Health, Johns Hopkins Bloomberg School of Public Health, Baltimore, Maryland, USA; University of Maryland School of Medicine

**Keywords:** COVID-19, SARS-CoV-2, health disparities, industrial livestock operations, seroprevalence

## Abstract

Industrial livestock operations (ILOs), particularly processing facilities, emerged as centers of coronavirus disease 2019 (COVID-19) outbreaks in spring 2020. Confirmed cases of COVID-19 underestimate true prevalence. To investigate the prevalence of antibodies against SARS-CoV-2, we enrolled 279 participants in North Carolina from February 2021 to July 2022: 90 from households with at least one ILO worker (ILO), 97 from high-ILO intensity areas (ILO neighbors [ILON]), and 92 from metropolitan areas (metro). More metro (55.4%) compared to ILO (51.6%) and ILON participants (48.4%) completed the COVID-19 primary vaccination series; the median completion date was more than 4 months later for ILO compared to ILON and metro participants, although neither difference was statistically significant. Participants provided a saliva swab we analyzed for SARS-CoV-2 IgG using a multiplex immunoassay. The prevalence of infection-induced IgG (positive for nucleocapsid and receptor binding domain) was higher among ILO (63%) than ILON (42.9%) and metro (48.7%) participants (prevalence ratio [PR], 1.38; 95% confidence interval [CI], 1.06 to 1.80; reference category ILON and metro combined). The prevalence of infection-induced IgG was also higher among ILO participants than among an Atlanta health care worker cohort (PR, 2.45; 95% CI, 1.80 to 3.33) and a general population cohort in North Carolina (PRs, 6.37 to 10.67). The infection-induced IgG prevalence increased over the study period. Participants reporting not masking in public in the past 2 weeks had higher infection-induced IgG prevalence (78.6%) than participants reporting masking (49.3%) (PR, 1.59; 95% CI, 1.19 to 2.13). Lower education, more people per bedroom, Hispanic/Latino ethnicity, and more contact with people outside the home were also associated with higher infection-induced IgG prevalence.

**IMPORTANCE** Few studies have measured COVID-19 seroprevalence in North Carolina, especially among rural, Black, and Hispanic/Latino communities that have been heavily affected. Antibody results show high rates of COVID-19 among industrial livestock operation workers and their household members. Antibody results add to evidence of health disparities related to COVID-19 by socioeconomic status and ethnicity. Associations between masking and physical distancing with antibody results also add to evidence of the effectiveness of these prevention strategies. Delays in the timing of receipt of COVID-19 vaccination reinforce the importance of dismantling vaccination barriers, especially for industrial livestock operation workers and their household members.

## INTRODUCTION

North Carolina is the second-largest hog, third-largest turkey, and fourth-largest broiler chicken-producing state (1). Animal slaughtering and processing workers have more than twice the rate of injury and illness (6.7 per 100 full-time equivalents), and animal production workers have close to twice the rate of injury and illness (5.2 per 100 full-time equivalents) compared to all United States workers (2.9 per 100 full-time equivalents), despite reporting exemptions and other factors likely resulting in injury and illness undercounts (2–5). Hog and poultry production have also been associated with a range of adverse health outcomes among nearby community residents, including respiratory health problems and infectious diseases (6–8). Since spring 2020, COVID-19 has become a health hazard associated with working at or living near industrial livestock operations (ILOs). Tens of thousands of cases of COVID-19 have been linked to work at meat and poultry processing facilities (9–11). Taylor et al. found an association between county livestock processing plants and county COVID-19 incidence, estimating that as many as 8% of United States cases through summer 2020 could be linked to processing plants (12). High numbers of COVID-19 cases have also been associated with food processing, food manufacturing, and agricultural workplaces more broadly (13). COVID-19 has disproportionately burdened low-income communities of color (14–17). Disparities by race and ethnicity are also evident among livestock processing and agricultural workers (10, 11, 13).

Given limited access to SARS-CoV-2 diagnostic testing, particularly for asymptomatic or mild COVID-19 cases, and limitations in diagnostic test reporting, SARS-CoV-2 antibody testing represents an attractive strategy to estimate COVID-19 prevalence, attack rates, and population immunity due to prior infection and/or vaccination (18, 20). SARS-CoV-2 antibodies in oral fluid (hereafter, salivary) have been shown to correspond to SARS-CoV-2 antibodies present in blood and to differentiate between PCR-confirmed cases and pre-COVID-19 samples with high sensitivity and specificity (19–21). Compared to blood collection, saliva collection is painless, safe, and readily conducted at home and mailed to a testing lab. However, saliva has been less frequently used for surveillance (20, 22).

We used a salivary multiplex immunoassay targeting IgG responses to SARS-CoV-2 nucleocapsid (N), receptor-binding domain (RBD), and spike (S) protein to differentiate between infection-induced versus infection- and/or vaccination-induced immune response. Infection induces antibodies against all proteins, while the mRNA (Pfizer-BioNTech, Moderna), Janssen (Johnson & Johnson), and Novavax vaccines currently approved for use in the United States induce RBD- and S-specific antibodies only (23). Accordingly, individuals testing positive for both SARS-CoV-2 N and RBD IgG likely experienced infection (24).

In this study, we measured salivary SARS-CoV-2 IgG prevalence in a cohort of North Carolina households enrolled in collaboration with REACH (Rural Empowerment Association for Community Help), a community group based in Duplin County, North Carolina. We aimed to (i) compare infection-induced IgG prevalence between participants living in households with at least one adult working at an industrial hog or poultry operation, meatpacking plant, or animal rendering plant (industrial livestock operation household group [ILO]), participants living near these facilities without any known occupational exposure to livestock (ILO neighbors [ILON]), and participants living in metropolitan areas of North Carolina (metro); (ii) identify risk factors for infection-induced IgG prevalence within our study population; and (iii) compare the infection-induced IgG prevalence between ILO participants and a cohort of other high-risk occupation workers sampled using the same assay, as well as a general population-representative cohort in North Carolina.

## RESULTS

### Participant characteristics.

A total of 279 individuals from 240 households (80 ILO, 80 ILON, and 80 metro) participated (Table 1). ILON participants were generally enrolled earliest, with a median interview date of 6 June 2021, followed by metro participants (median, 18 August 2021), and ILO participants (median, 9 February 2022). Overall, ILON participants were older than ILO and metro participants. Roughly half of ILO and ILON participants were female, compared to 72.8% of metro participants. Most participants in all groups were Black; more metro participants were White and fewer were Hispanic/Latino compared to ILO and ILON participants. Educational attainment differed between groups: most ILO participants had a high school education or lower, while most metro participants had post-high school education. Most participants lived in homes with one or fewer household members per bedroom, although ILO participants reported more household members per bedroom than ILON and metro participants. The majority of participants reported that their household’s primary health care provider was a private doctor or clinic. However, more ILON (15.5%) than ILO (13.3%) and metro (8.7%) participants reported not having health insurance. More than twice as many ILO participants (91.1%) reported working in person compared to ILON (42.3%) and metro (41.3%) participants.

**TABLE 1:** Participant characteristics by study group, North Carolina, 2021 to 2022^*a*^

Characteristic	ILO (*n* = 90) (80 households)	ILON (*n* = 97) (80 households)	Metro (*n* = 92) (80 households)
Sampling date (mo/day/yr), median (range)	2/9/2022 (4/2/2021–7/18/2022)	6/6/2021 (3/8/2021–6/3/2022)	8/18/2021 (2/23/2021–6/7/2022)
Age in yrs, median (range)	41.5 (13–67)	50 (5–83)	37 (9–74)
Gender, *n* (%)			
Female	47 (52.2)	54 (55.7)	67 (72.8)
Male	43 (47.8)	42 (43.3)	25 (27.2)
No response	0 (0)	1 (1)	0 (0)
Race/ethnicity, *n* (%)			
Black/African American	79 (87.8)	83 (85.6)	75 (81.5)
Hispanic/Latino	8 (8.9)	11 (11.3)	4 (4.3)
White	1 (1.1)	1 (1)	8 (8.7)
Both Black and White	0 (0)	1 (1)	2 (2.2)
Asian American	0 (0)	0 (0)	2 (2.2)
Other or no response	2 (2.2)	1 (1)	1 (1.1)
Education, *n* (%)			
High school diploma/GED or less	65 (72.2)	53 (54.6)	37 (40.2)
Post-high school	25 (27.8)	42 (43.3)	53 (57.6)
No response	0 (0)	2 (2.1)	2 (2.2)
Household members, mean (SD)	2.7 (1.4)	2.1 (1.3)	2.2 (1.2)
Household members per bedroom, mean (SD)	1 (0.5)	0.8 (0.4)	0.9 (0.5)
Primary healthcare provider, *n* (%)^*b*^			
Private doctor’s office or clinic	56 (62.2)	55 (56.7)	54 (58.7)
Urgent care	15 (16.7)	23 (23.7)	17 (18.5)
Emergency room	14 (15.6)	11 (11.3)	13 (14.1)
Hospital	10 (11.1)	13 (13.4)	8 (8.7)
Free clinic	6 (6.7)	7 (7.2)	5 (5.4)
Company clinic, doctor, or nurse	6 (6.7)	1 (1)	5 (5.4)
Do not use medical care	2 (2.2)	1 (1)	0 (0)
Health insurance, *n* (%)^*b*^			
Company health insurance plan	30 (33.3)	33 (34)	56 (60.9)
Public health insurance	34 (37.8)	35 (36.1)	17 (18.5)
Private health insurance	15 (16.7)	16 (16.5)	10 (10.9)
No health insurance	12 (13.3)	15 (15.5)	8 (8.7)
Other or no response	2 (2.2)	0 (0)	1 (1.1)
Work or attend school or childcare outside the home, *n* (%)	85 (94.4)	44 (45.4)	41 (44.6)
Work (in person) only	82 (91.1)	41 (42.3)	37 (40.2)
Attend school only	2 (2.2)	3 (3.1)	3 (3.3)
Work and attend school	0 (0)	0 (0)	1 (1.1)
Attend childcare	1 (1.1)	0 (0)	0 (0)
Do not work or attend school or childcare outside the home, *n* (%)	5 (5.6)	53 (54.6)	51 (55.4)

a ILO, study participants living in a household with at least one adult working at an industrial hog or poultry operation, meatpacking plant, or animal rendering plant; ILON, participants living near these facilities without any known occupational exposure to livestock; metro, participants living in metropolitan areas.

b Participants could select more than one option, so percentages may not sum to 100.

### COVID-19 vaccination over time by study group.

**FIG 1:**
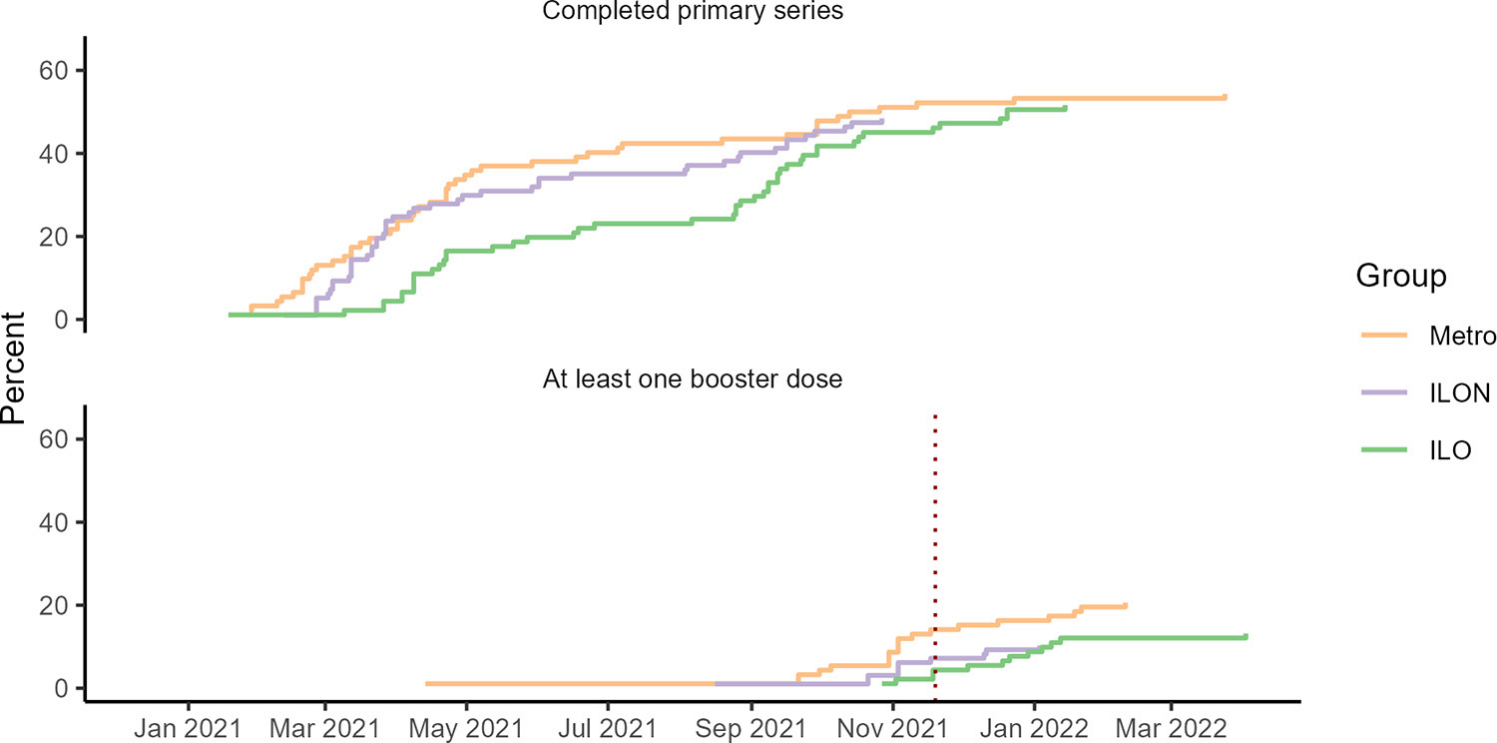
COVID-19 vaccination over time by study group^a^, North Carolina, 2021 to 2022. Origin is the date of the first FDA emergency use authorization for a vaccine against COVID-19 (Pfizer-BioNTech), 11 December 2020, and the dotted line is the date on which the CDC expanded eligibility for a booster shot to all adults, 29 November 2021 (54). ILO, study participants living in a household with at least one adult working at an industrial hog or poultry operation, meatpacking plant, or animal rendering plant; ILON, participants living near these facilities without any known occupational exposure to livestock; metro, participants living in metropolitan areas.

More metro participants (20.7%) were up to date with COVID-19 vaccines, receiving all doses in the primary series and at least one booster, than ILO (13.2%) or ILON (10.3%) participants, although differences were not statistically significant (see Table S1 in the supplemental material). More metro participants (55.4%) also completed the COVID-19 primary vaccination series, receiving either a first dose and second dose of the Pfizer, Moderna, or Novavax vaccines or a single dose of the Johnson & Johnson vaccine, than ILO (51.6%) or ILON (48.5%) participants, although differences were not statistically significant (Table S1). There was no statistically significant difference in time to primary series completion or becoming up to date between the study groups (*P* values for 3-group log-rank test, <0.4 and <0.1, respectively). However, among participants who received a booster dose, the median date of receiving that booster dose was later for ILO (19 December 2021) than for ILON (3 November 2021) and metro (3 November 2021) participants, and the same pattern held for primary series completion (Fig. 1).

10.1128/msphere.00522-22.3 TABLE S1 COVID-19 vaccination by study group compared to North Carolina and Unite States by quartile of interview or follow-up call dates. *^a^*CDC estimates from Our World in Data visualization (37). REACH, our study population and collaboration with the Rural Empowerment Association for Community Help (REACH); ILO, study participants living in a household with at least one adult working at an industrial hog or poultry operation, meatpacking plant, or animal rendering plant; ILON, participants living near these facilities without any known occupational exposure to livestock; metro, participants living in metropolitan areas. Participants who reported vaccination but did not provide a corresponding date are included as by 18 July 2022, the final date of interviews and follow-up calls. Download Table S1, DOCX file, 0.03 MB. Copyright © 2023 Gigot et al. 2023 Gigot et al. https://creativecommons.org/licenses/by/4.0/ This content is distributed under the terms of the Creative Commons Attribution 4.0 International license.

### SARS-CoV-2 IgG antibody and self-reported COVID-19 outcomes.

Most participant saliva samples had SARS-CoV-2 IgG antibodies (Table 2). The prevalence of infection-induced IgG (positive for both N and RBD) was higher among ILO (63%) than among ILON (42.9%) and metro (48.7%) participants (prevalence ratio [PR], 1.38; 95% confidence interval [CI], 1.06 to 1.8; ref. ILON and metro combined). The prevalence of infection- and/or vaccination-induced IgG (positive for RBD) was similar among ILO (78.1%), ILON (63.1%), and metro participants (77.6%). Significantly more ILO participants reported at least one and at least two COVID-19 symptoms than ILON and metro participants (25). Fewer participants reported thinking they had COVID-19 than had infection-induced IgG. Even fewer participants reported they had ever tested positive for SARS-CoV-2, with the highest proportion among metro participants (21.1%), followed by ILON (11.9%) and ILO (13.7%) participants.

**TABLE 2:** SARS-CoV-2 IgG antibody and self-reported COVID-19 prevalence, prevalence ratios (PR), and 95% confidence intervals (CI) by study group, North Carolina, 2021 to 2022^*a*^

Outcome	Number positive/total (%)	Crude PR (95% CI)
Antibody assay results		
SARS-CoV-2 infection-induced IgG (positive for both N and RBD)		
ILO vs:	46/73 (63)	
ILON (reference)	36/84 (42.9)	1.47 (1.05–2.07)
Metro (reference)	37/76 (48.7)	1.29 (0.95–1.76)
ILON and metro (reference)	73/160 (45.6)	1.38 (1.06–1.8)
SARS-CoV-2 infection- and/or vaccination- induced IgG (positive for RBD)		
ILO vs:	57/73 (78.1)	
ILON (reference)	53/84 (63.1)	1.24 (1–1.53)
Metro (reference)	59/76 (77.6)	1.01 (0.85–1.19)
ILON and metro (reference)	112/160 (70)	1.12 (0.95–1.31)
Self-reported COVID-19		
At least one symptom of COVID-19^*b*^		
ILO vs:	48/73 (65.8)	
ILON (reference)	40/84 (47.6)	1.38 (1.03–1.85)
Metro (reference)	39/76 (51.3)	1.28 (0.96–1.71)
ILON and metro (reference)	79/160 (49.4)	1.33 (1.05–1.7)
At least two symptoms of COVID-19^*b*^		
ILO vs:	40/73 (54.8)	
ILON (reference)	29/84 (34.5)	1.59 (1.08–2.33)
Metro (reference)	35/76 (46.1)	1.19 (0.85–1.67)
ILON and metro (reference)	64/160 (40)	1.37 (1.02–1.84)
Ever thought you had COVID-19		
ILO vs:	19/73 (26)	
ILON (reference)	16/84 (19)	1.37 (0.76–2.47)
Metro (reference)	16/76 (21.1)	1.24 (0.67–2.28)
ILON and metro (reference)	32/160 (20)	1.3 (0.78–2.17)
Ever tested positive for SARS-CoV-2		
ILO vs:	10/73 (13.7)	
ILON (reference)	10/84 (11.9)	0.96 (0.45–2.02)
Metro (reference)	16/76 (21.1)	0.67 (0.34–1.29)
ILON and metro (reference)	26/160 (16.2)	0.78 (0.42–1.45)

a ILO, study participants living in a household with at least one adult working at an industrial hog or poultry operation, meatpacking plant, or animal rendering plant; ILON, participants living near these facilities without any known occupational exposure to livestock; metro, participants living in metropolitan areas.

b Listed by CDC: fever or chills; cough; shortness of breath or difficulty breathing; lack of energy or general tired feeling; muscle or body aches; headache; new loss of taste or smell; sore throat, congestion, or runny nose; feeling sick to your stomach or vomiting, diarrhea; abdominal pain; skin rash (25); since 1 February 2020.

### SARS-CoV-2 infection-induced IgG prevalence by participant characteristics.

The proportion of participants with salivary SARS-CoV-2 infection-induced IgG increased over the study period (Table 3). Several participant demographic characteristics and infection prevention behaviors were associated with infection-induced IgG prevalence. The strongest association was for participants who reported generally wearing a mask in public in the past 2 weeks. Participants who reported not wearing a mask had significantly higher infection-induced IgG prevalence (78.6%) than participants who reported wearing a mask (49.3%) (PR, 1.59; 95% CI, 1.19 to 2.13). Participants with greater than a high school education had significantly lower infection-induced IgG prevalence (38.1%) than participants with a high school education or less (60.2%) (PR, 0.63; 95% CI, 0.48 to 0.84). Participants who lived in households with more than one person per bedroom had significantly higher infection-induced IgG prevalence (69.4%) than participants in households with one person or fewer per bedroom (46.4%) (PR, 1.50; 95% CI, 1.15 to 1.95). Hispanic/Latino participants had significantly higher infection-induced IgG prevalence (72.7%) than Black participants (49.7%) (PR, 1.46; 95% CI, 1.1 to 1.94). Only 16.7% of White participants had infection-induced IgG. Reduced contact with people outside the home was associated with reduced infection-induced IgG prevalence.

**TABLE 3:** SARS-CoV-2 infection-induced IgG prevalence, prevalence ratios (PR), and 95% confidence intervals (CI) by participant characteristics, North Carolina, 2021 to 2022

Characteristic	Number positive/total (%)	Crude PR (95% CI)
Sampling date, quartiles (mo/day/yr)		
2/23/21–4/30/21	14/59 (23.7)	Reference
4/30/21–9/6/21	23/58 (39.7)	1.67 (0.87–3.22)
9/6/21–3/11/22	37/58 (63.8)	2.69 (1.49–4.84)
3/11/22–7/18/22	45/58 (77.6)	3.27 (1.85–5.79)
Demographic characteristics		
Age in yrs, quartiles		
5–27	27/58 (46.6)	Reference
27–41	28/58 (48.3)	1.04 (0.71–1.51)
42–55	35/57 (61.4)	1.32 (0.93–1.86)
55–83	27/57 (47.4)	1.02 (0.68–1.54)
Sex		
Female	72/139 (51.8)	Reference
Male	47/94 (50)	0.97 (0.76–1.23)
Race/ethnicity		
Black	99/199 (49.7)	Reference
Hispanic/Latino	16/22 (72.7)	1.46 (1.1–1.94)
White	1/6 (16.7)	0.34 (0.05–2.14)
Other	3/6 (50)	1.01 (0.45–2.27)
Education		
≤High school	80/133 (60.2)	Reference
>High school	37/97 (38.1)	0.63 (0.48–0.84)
Household members per bedroom		
≤1 person per bedroom	84/181 (46.4)	Reference
>1 person per bedroom	34/49 (69.4)	1.50 (1.15–1.95)
Work in person outside the home		
No	44/97 (45.4)	Reference
Yes	75/136 (55.1)	1.22 (0.93–1.59)
Work in meatpacking		
No	109/257 (42.4)	Reference
Yes	10/23 (43.5)	0.98 (0.61–1.57)
Infection prevention behaviors		
Reduced contact with people outside your home		
Yes, all household members	89/178 (50)	Reference
Yes, some but not all household members	12/17 (70.6)	1.41 (1–2)
No	13/18 (72.2)	1.44 (1.04–2)
Avoiding or cancelling travel or vacation plans		
Yes	85/170 (50)	Reference
No	33/61 (54.1)	1.08 (0.82–1.43)
Wearing a mask when out in public		
Yes	108/219 (49.3)	Reference
No	11/14 (78.6)	1.59 (1.19–2.13)
Washing hands/using hand sanitizer more frequently		
Yes	112/221 (50.7)	Reference
No	6/10 (60)	1.18 (0.7–1.99)
COVID-19 symptoms and health history		
Ever tested positive for SARS-CoV-2		
No	45/100 (45)	Reference
Yes	31/36 (86.1)	7.58 (2.74–20.9)^*a*^
At least one symptom of COVID-19^*b*^		
No	52/106 (49.1)	Reference
Yes	67/127 (52.8)	1.08 (0.83–1.39)
At least two symptoms of COVID-19^*b*^		
No	62/129 (48.1)	Reference
Yes	57/104 (54.8)	1.14 (0.88–1.47)
Ever thought you had COVID-19		
No	77/173 (44.5)	
Yes	36/51 (70.6)	1.59 (1.24–2.03)
Fever with cough or fever with sore throat at the same time^*c*^ (past yr)		
No	89/190 (46.8)	Reference
Yes	28/41 (68.3)	1.46 (1.12–1.89)
At least one chronic condition associated with severe illness from COVID-19^*d*^		
No	64/162 (39.5)	Reference
Yes	55/118 (46.6)	1.25 (0.97–1.61)

a Odds ratio (OR) because log-binomial model failed to converge.

b Listed by CDC: fever or chills; cough; shortness of breath or difficulty breathing; lack of energy or general tired feeling; muscle or body aches; headache; new loss of taste or smell; sore throat, congestion, or runny nose; feeling sick to your stomach or vomiting, diarrhea; abdominal pain; skin rash (25); since February 1, 2020.

c CDC definition of influenza-like illness (26).

d Listed by CDC: diabetes, cardiovascular disease, hypertension, immunocompromised condition, autoimmune disease, cancer, chronic kidney disease, asthma, COPD, other chronic lung disease, sickle cell anemia, depression, other mental health disorder (27).

Participants’ COVID-19 symptoms and health history also corresponded with SARS-CoV-2 IgG. Participants who reported ever testing positive for SARS-CoV-2 had significantly higher infection-induced IgG prevalence (86.1%) than those who reported never testing positive (45%) (odds ratio [OR], 7.58; 95% CI, 2.74 to 20.9). While participants’ report of at least one or at least two COVID-19 symptoms listed by the CDC was not associated with infection-induced IgG prevalence, participants who thought they had COVID-19 had higher infection-induced IgG prevalence (70.6%) than participants who thought they had not (44.5%) (PR, 1.59; 95% CI, 1.24 to 2.03). Participants who reported fever plus cough or sore throat had higher infection-induced IgG prevalence (68.3%) than those who did not (46.8%) (PR, 1.46; 95% CI, 1.12 to 1.89) (26). Almost half (46.6%) of participants who reported a chronic medical condition listed by the CDC as increasing the risk of getting very sick with COVID-19 had infection-induced IgG (27).

The highest correlation between factors associated with SARS-CoV-2 infection-induced IgG was between date quartile and vaccination status (Cramer’s V-test value, 0.42), followed by date and study group (0.39), group and education level (0.25), group and household members per bedroom (0.23), and date and level of contact with people outside the home (0.23) (Fig. S1).

10.1128/msphere.00522-22.1 FIG S1 Correlation matrix (Cramer’s V correlation values) between factors associated with SARS-CoV-2 infection-induced IgG prevalence, categorized as in Table 3. Cramer’s V test is based on Pearson’s Chi-square statistic and ranges between 0 and 1, with more correlated nominal variables having higher values. Download FIG S1, TIF file, 2.5 MB. Copyright © 2023 Gigot et al. 2023 Gigot et al. https://creativecommons.org/licenses/by/4.0/ This content is distributed under the terms of the Creative Commons Attribution 4.0 International license.

### SARS-CoV-2 infection-induced IgG prevalence compared to other southern United States cohorts.

**FIG 2:**
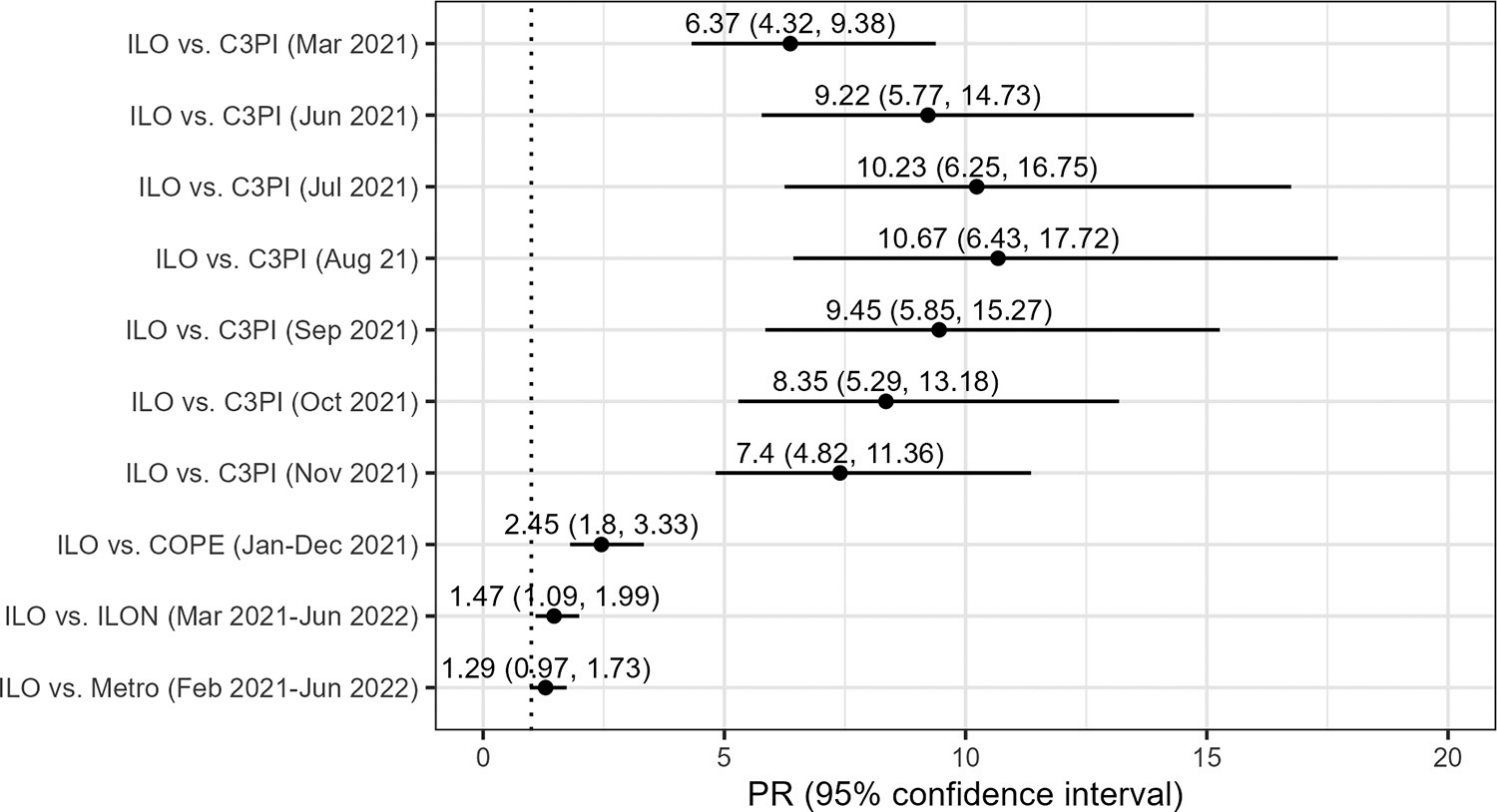
SARS-CoV-2 infection-induced IgG prevalence ratios (PRs) among North Carolina ILO household group participants (measured April 2021 to July 2022) compared to other southern United States reference populations. ILO, study participants living in a household with at least one adult working at an industrial hog or poultry operation, meatpacking plant, or animal rendering plant; ILON, participants living near these facilities without any known occupational exposure to livestock; metro, participants living in metropolitan areas; C3PI, the MURDOCK Cabarrus County COVID-19 Prevalence and Immunity (C3PI) Study cohort, representative of the general population of Cabarrus County, North Carolina; COPE, the COVID-19 Prevention in Emory Healthcare Personnel (COPE) Study cohort.

The prevalence of SARS-CoV-2 infection-induced IgG was significantly higher in the ILO group of our study population than two other southern United States cohorts sampled at times overlapping the interview and sampling dates of our study. Infection-induced IgG prevalence was significantly higher in the ILO group of our study population sampled between March 2021 and July 2022 (63%) than the COVID-19 Prevention in Emory Healthcare Personnel (COPE) Study cohort sampled between January and December 2021 (23.2%) using the same salivary multiplex assay (PR, 2.45; 95% CI, 1.80 to 3.33) (M. H. Collins and C. D. Heaney, correspondence) (28, 29) (Fig. 2). Infection-induced IgG prevalence was also significantly higher in the ILO group of our study population (63%) than the MURDOCK Cabarrus County COVID-19 Prevalence and Immunity (C3PI) Study cohort, aiming to be representative of the general population of Cabarrus County, North Carolina, sampled March and monthly June through November 2021, using blood testing with the Abbott Alinity N IgG assay (5.9% to 9.9%; PR range, 6.37 to 10.67) (L. K. Newby and D. Wixted, correspondence) (30).

## DISCUSSION

We measured salivary SARS-CoV-2 IgG prevalence among more than 200 participants living in North Carolina, 94% of whom were Black or Hispanic/Latino, underrepresented groups in SARS-CoV-2 seroprevalence surveys. To our knowledge, this study presents the first estimates of SARS-CoV-2 infection-induced antibody prevalence among industrial livestock operation (ILO) workers and their household members, which we observed to be high (63%) compared to participants with no household members working at an industrial livestock operation (45.6%) (Table 2). This is consistent with research connecting the agricultural sector and meatpacking facilities with COVID-19 transmission among workers and research connecting meatpacking facilities with transmission in nearby communities (10, 12, 13, 31). However, neither work at a meatpacking facility nor work in person outside the home was associated with elevated prevalence of SARS-CoV-2 infection-induced IgG in our study population (Table 3), and participants living in areas of high industrial livestock operation intensity did not have a higher prevalence (42.9%) than metropolitan-area participants (48.7%) (Table 2). This may be due to the relatively small number of meatpacking workers (*n* = 23) and relatively large number of participants with other high-COVID-19-risk jobs and risk factors (e.g., low-income and communities of color). Another contributing factor could be case rate convergence over time between high-intensity livestock operation areas and metropolitan areas. As the prevalence of COVID-19 increased in summer and fall 2020, the importance of any single transmission route decreased; also, if many meatpacking workers and nearby residents were infected earlier on, those communities might have a greater rate of at least temporary immunity (31). Our group definitions of ILO, ILON, and metro also collapse many differences that have been connected to exposure to SARS-CoV-2 and COVID-19: time, age, sex, race/ethnicity, education, and urbanicity, among others (14–16).

Self-reported symptoms of COVID-19 were also higher in the ILO than in the combined ILON and metro groups (Table 2). This could be because of more COVID-19 cases, though some contribution could also be from other health effects related to industrial livestock production and processing work. Hog and poultry production work have been associated with respiratory and infectious disease broadly, and processing work has also been associated with respiratory, infectious, and skin disease, which overlap with almost all COVID symptoms listed by the CDC (6, 25, 32, 33). While 51% of participants overall had SARS-CoV-2 infection-induced IgG, less than half reported thinking they had COVID-19, and only 15.4% reported ever testing positive for COVID-19 (Tables 2 and 3). Of participants who tested positive for infection-induced IgG, 68% did not think they had COVID-19 (Table 3). This is a higher proportion than the estimated prevalence of asymptomatic infections among people with confirmed COVID-19, 40 to 45% (34). Participants might have attributed any symptoms to other health issues, including some related to ILO work or residence near ILOs. The lower percentage of people ever testing positive for COVID-19 even compared to the low percentage of people who thought they had COVID-19 also underlines the importance of accessible COVID-19 diagnostic testing.

A higher proportion of metro compared to ILO or ILON participants completed the primary vaccination series and received at least one booster, although the difference between groups was not statistically significant, and the groups had similar SARS-CoV-2 infection- and/or vaccination-induced IgG (positive for RBD) (Fig. 1, Table 2). Among participants who completed the primary vaccination series, the median date of completion was later for ILO than ILON and metro participants, and the same pattern held for booster doses (Fig. 1). Although the differences in vaccination timing between groups were not statistically significant, these delays are notable because of the spread of the more-contagious SARS-CoV-2 Delta and Omicron variants in summer and winter 2021, respectively (35). Because a greater proportion of ILO participants had a high school education or less, our results are also consistent with evidence of vaccination disparities by social class and with evidence that vaccination coverage increased most during spring and summer 2021 among people with lower education and income (36).

COVID-19 vaccination rates in our study population were lower than the United States and North Carolina general populations. The proportion of our study population (ILO, ILON, and metro combined) who completed the initial vaccination protocol (52%) was lower than the proportion of North Carolina (62.9%) and United States residents (67.2%) vaccinated at the last date of follow-up, 18 July 2022 (37) (Table S1). However, participants may have become vaccinated after their initial or follow-up call; the proportion of participants who had completed the initial vaccination protocol by the median initial or follow-up call date was 50.7%, closer to the proportion of North Carolina (57.3%) and the United States (62.6%) at that date, 10 January 2022. The proportion of our cohort who received at least one booster dose was also lower than that for North Carolina and the United States at the end of the study, but closer at the median follow-up date (37). Although our modest sample size and timing of initial and follow-up calls complicate comparison to North Carolina and the United States, our results support the importance of dismantling vaccination barriers, especially for ILO workers and their household members and rural communities.

The proportion of participants with SARS-CoV-2 infection-induced IgG prevalence increased over the course of the study (Table 3). This is consistent with the spread of the virus over time and trends in other seroprevalence surveys (24, 38). Among health behaviors assessed, wearing a mask had the highest protective effect (Table 3). Mask use was also similar across study groups (Table S2). This is consistent with cohort, ecological, and modeling studies on the efficacy of masks for COVID-19 protection (39). Reporting mask use could also be an indicator of other modifiable and nonmodifiable risk factors. We did not ask about the frequency of mask use overall, mask use in particular contexts that might be higher-risk transmission settings, or about the type(s) of masks participants used, all of which affect the relationship between mask use and SARS-CoV-2 exposure.

10.1128/msphere.00522-22.4 TABLE S2 Self-reported infection prevention behaviors and health history by study group, North Carolina, 2021 to 2022. *^a^P* value associated with the Chi-square test statistic. *^b^*CDC definition of influenza-like illness (26). ILO, study participants living in a household with at least one adult working at an industrial hog or poultry operation, meatpacking plant, or animal rendering plant; ILON, participants living near these facilities without any known occupational exposure to livestock; metro, participants living in metropolitan areas. Download Table S2, DOCX file, 0.03 MB. Copyright © 2023 Gigot et al. 2023 Gigot et al. https://creativecommons.org/licenses/by/4.0/ This content is distributed under the terms of the Creative Commons Attribution 4.0 International license.

Education level and ethnicity were also associated with SARS-CoV-2 infection-induced IgG. A study of the joint effects of socioeconomic position estimated by education level, race/ethnicity, and gender on COVID-19 mortality among working-age adults found the same trends of higher COVID-19 mortality for low versus high socioeconomic position for adults and for Black and Hispanic/Latino versus White adults (40). Our results are also consistent with elevated rates of infection-induced seroprevalence among Hispanic/Latino and Black compared with White blood donors across the United States (24) and elevated infection-induced seroprevalence among Hispanic/Latino and Black North Carolina residents in surveillance based on hospital remnant blood samples (16), as well as with disproportionate numbers of cases and deaths among Hispanic/Latino, Native American, and Black communities (14). People with fewer socioeconomic resources are less able to use different strategies to avoid exposure to SARS-CoV-2 and more likely to work in crowded occupations or occupations with contact with the public (40). Under racialized capitalism, Hispanic/Latino, indigenous, and Black workers face occupational disadvantages even within specific jobs (40, 41).

We found that participants with more than one person per household bedroom had higher prevalence of SARS-CoV-2 infection-induced IgG (Table 3). Living in more crowded conditions could increase exposures to SARS-CoV-2 from household members. Level of contact with people outside the home was also associated with higher infection-induced IgG prevalence (Table 3). A systematic review of observational studies of SARS-CoV-2 and the betacoronaviruses that cause severe acute respiratory syndrome (SARS-CoV-1) and Middle East respiratory syndrome (MERS) found reduced transmission of viruses with physical distancing of 1 meter or more as well as with face mask usage, consistent with our results (42).

Participants’ COVID-19 health history and symptoms also corresponded with SARS-CoV-2 IgG prevalence. Close to 90% of participants who reported ever testing positive for SARS-CoV-2 had infection-induced IgG (positive for both N and RBD), compared to 45% of participants who did not report ever testing positive (Table 3). N IgG is useful for determining response to infection because these antibodies are produced in the immune response to infection and not in the response to vaccines currently approved for use in the United States (23). However, a limitation is that the N IgG half-life in the body is generally shorter than that of RBD and S IgG (43). Participants who reported testing positive for SARS-CoV-2 but tested negative for N IgG could have been infected longer ago and have levels of N IgG below the positivity cutoff. Participants who reported thinking they had COVID-19 and participants who reported influenza-like illness had a higher prevalence of infection-induced IgG than participants who did not, although there was a similar prevalence of infection-induced IgG among participants who reported at least one or at least two symptoms of COVID-19 compared to those who did not (Table 3). This is consistent with relatively high proportions of asymptomatic infections (34), overlap between COVID-19 and other health condition symptoms, and limited durability of N IgG response.

We found prevalence of SARS-CoV-2 infection-induced IgG higher than in comparable cohorts. Infection-induced IgG prevalence in the ILO group of our study population was more than twice that in Emory University’s COPE cohort of Atlanta, Georgia, health care workers sampled from January to December 2021 using the same salivary multiplex assay (M. H. Collins and C. D. Heaney, correspondence) (28, 29) (Fig. 2). Health care workers are at higher risk for COVID-19 than the general population (44, 45). The high infection-induced IgG among ILO participants compared to workers in another high-COVID-19-risk occupation sampled with the same assay during an overlapping time period underlines the high exposures among North Carolina livestock operation workers and their household members. The infection-induced IgG prevalence among ILO participants was more than five times the highest prevalence observed in March and monthly from June through November 2021 in Duke University’s C3PI Cabarrus County, North Carolina, general population-representative cohort (L. K. Newby and D. Wixted, correspondence) (30) (Fig. 2). Infection-induced IgG prevalence was also higher in the ILO group of our study population (63%) than nationwide serology estimates. A study of blood donations estimated infection-induced seroprevalence to be 28.8% overall in December 2021, higher among Hispanic (40.2%) and Black (32.5%) donors and donors living in the South (33.5%) during the same time period (24). The infection-induced IgG prevalence in our cohort was also generally higher than estimates using residual data from commercial labs across the United States weighted by age, sex, and metropolitan status, which ranged from 20.8% to 57.7% nationally and 22.5% to 52% in North Carolina during the 16 sampling periods that overlapped our study period (35, 38) (Fig. S2).

10.1128/msphere.00522-22.2 FIG S2 SARS-CoV-2 infection-induced IgG prevalence over time by cohort; the ILO, ILON, and metro groups are split into participants sampled before versus after the median sampling date, 6 September 2021, for better comparability with other cohorts over time. ILO, study participants living in a household with at least one adult working at an industrial hog or poultry operation, meatpacking plant, or animal rendering plant; ILON, participants living near these facilities without any known occupational exposure to livestock; metro, participants living in metropolitan areas; COPE, the COVID-19 Prevention in Emory Healthcare Personnel (COPE) Study cohort; C3PI, the MURDOCK Cabarrus County COVID-19 Prevalence and Immunity (C3PI) Study cohort, representative of the general population of Cabarrus County, North Carolina; CDC NC, North Carolina infection-induced antibody seroprevalence estimates from CDC commercial laboratory surveys, downloaded from https://covid.cdc.gov/covid-data-tracker/#national-lab (35). Download FIG S2, TIF file, 2.7 MB. Copyright © 2023 Gigot et al. 2023 Gigot et al. https://creativecommons.org/licenses/by/4.0/ This content is distributed under the terms of the Creative Commons Attribution 4.0 International license.

An important consideration for interpreting our results is our non-population-representative snowball sampling strategy. Participants were volunteers recruited primarily from social networks of community organizers with our partner community organization and might differ in several ways from the North Carolina population in general. Another consideration is our enrollment period from February 2021 to July 2022, which included changing recommendations on COVID-19 vaccination and boosters, as well as increasing cases due to the more contagious Delta and Omicron variants. Vaccination, exposure, and treatment options varied for participants over the course of enrollment, and differences over time may obscure differences by study group or participant characteristics. The long enrollment period also complicates comparisons with other southern United States cohorts and CDC nationwide studies (Fig. S2).

Our study also has limitations associated with antibody test characteristics. The half-life of SARS-CoV-2 N IgG using our assay was about 64 days, compared to RBD at about 100 days (43). This waning, especially of N-specific antibodies, may have caused us to underestimate infection-induced SARS-CoV-2 exposure. Variability in commercial anti-N antibody assay performance suggests faster waning of N-specific antibodies may be related to assay characteristics rather than biological waning (46–48). We used a quantitative antibody assay, which generally perform better than lateral flow assays (48). Assays targeting RBD generally have the slowest decay and loss of sensitivity over time, followed by S, followed by N; our multiplex approach incorporating both RBD and N may have allowed us to maintain sensitivity with a lower N cutoff (43, 48). Variability in anti-N antibody assay sensitivity over time is also a limitation when comparing our SARS-CoV-2 infection-induced antibody prevalence estimates to other studies. Neighbors et al. used the Abbott Alinity N IgG assay to estimate seroprevalence in the C3PI Cabarrus County cohort, which Owusu-Boaitey found to be one of the poorer-performing assays (30, 48). If the Abbott Alinity N IgG assay had a lower sensitivity over time compared to our assay, prevalence ratios comparing our ILO participants to the C3PI cohort could be overestimated. Another consideration is emerging evidence that N seroconversion is less likely to occur postinfection in vaccinated individuals (49). Most of the COPE cohort completed the primary COVID-19 vaccination series (81.4%) compared to about half of ILO participants (51.6%) and our study population overall (52%) (Table S3). Prevalence ratios comparing ILO to COPE participants might also be overestimated. However, we found higher infection-induced IgG prevalence among both vaccinated ILO participants (72%) than vaccinated COPE participants (18%) and among unvaccinated ILO participants (56%) than unvaccinated COPE participants (36%), suggesting that elevated prevalence ratios among ILO compared to COPE participants are robust to the effect of vaccination on N seroconversion (Table S4).

10.1128/msphere.00522-22.5 TABLE S3 Selected participant characteristics by study group compared to other southern United States reference populations. *^a^*The number of samples with SARS-CoV-2-specific N IgG results does not necessarily match the size of the study population group. Not all REACH samples had valid results; COPE and C3PI N IgG results are only shown for participants with valid results from the latest follow-up visit (28). *^b^*Participants could select more than one option. REACH, our study population and collaboration with the Rural Empowerment Association for Community Help; ILO, study participants living in a household with at least one adult working at an industrial hog or poultry operation, meatpacking plant, or animal rendering plant; ILON, participants living near these facilities without any known occupational exposure to livestock; metro, participants living in metropolitan areas; COPE, the COVID-19 Prevention in Emory Healthcare Personnel (COPE) Study cohort; C3PI, the MURDOCK Cabarrus County COVID-19 Prevalence and Immunity (C3PI) Study cohort, representative of the general population of Cabarrus County, North Carolina; —, data not available. Download Table S3, DOCX file, 0.03 MB. Copyright © 2023 Gigot et al. 2023 Gigot et al. https://creativecommons.org/licenses/by/4.0/ This content is distributed under the terms of the Creative Commons Attribution 4.0 International license.

10.1128/msphere.00522-22.6 TABLE S4 SARS-CoV-2 infection-induced IgG prevalence by study group compared to other southern reference populations, stratified by participant vaccination status at sampling date. *^a^*Categories are not mutually exclusive. REACH, our study population and collaboration with the Rural Empowerment Association for Community Help; ILO, study participants living in a household with at least one adult working at an industrial hog or poultry operation, meatpacking plant, or animal rendering plant; ILON, participants living near these facilities without any known occupational exposure to livestock; metro, participants living in metropolitan areas; COPE, the COVID-19 Prevention in Emory Healthcare Personnel (COPE) Study cohort; C3PI, the MURDOCK Cabarrus County COVID-19 Prevalence and Immunity (C3PI) Study cohort, representative of the general population of Cabarrus County, North Carolina; —, data not available. Download Table S4, DOCX file, 0.02 MB. Copyright © 2023 Gigot et al. 2023 Gigot et al. https://creativecommons.org/licenses/by/4.0/ This content is distributed under the terms of the Creative Commons Attribution 4.0 International license.

Our findings show high rates of SARS-CoV-2 infection-induced IgG in a predominantly rural, Black, and Hispanic/Latino North Carolina study population, especially among industrial livestock operation workers and their household members. We add to reports of high numbers of cases associated with meatpacking facilities early in the course of the COVID-19 pandemic and to evidence of health disparities in exposure to SARS-CoV-2 by socioeconomic position. Delays in the timing of receipt of COVID-19 vaccination reinforce the importance of dismantling vaccination barriers, especially for industrial livestock operation workers and their household members. Associations between masking and physical distancing with antibody results also add to evidence of the effectiveness of these prevention strategies.

## MATERIALS AND METHODS

### Study design and participants.

This study was designed and conducted in partnership between Johns Hopkins Bloomberg School of Public Health (JHSPH) and REACH. Data were collected by REACH community organizers and researchers from JHSPH. Using a snowball sampling approach, REACH community organizers recruited ILO, ILON, and metro households in North Carolina. All households had at least one adult (≥18 years old) enrolled into the study. In addition to the one adult, all household members of any age were eligible to be enrolled. Eligibility criteria for all groups also included the ability to understand spoken English or Spanish and access to a household phone or mobile device and refrigerator. The JHSPH Institutional Review Board (IRB) approved this study (IRB00014420).

### Questionnaire data and saliva sample collection.

Before participation, adult participants provided oral consent. For children 0 to 6 years old, a parent or legal guardian provided permission, oral assent, and questionnaire responses. For children 7 to 17 years old, a parent or legal guardian provided permission for the child, and the child provided oral assent and questionnaire responses, with parents answering some questions as appropriate (e.g., health history). Recruitment, consent, questionnaire administration, and saliva self-collection were primarily conducted remotely via video or phone calls, without physical contact between the study team and participants. After consent, all enrolled households received a study package containing all materials for saliva self-collection, self-collection procedure information, and packaging materials via REACH drop-off or direct shipping from JHSPH.

Questionnaire responses were recorded and training and supervision of biospecimen self-collection were provided during the same video or phone call. Participants and parents or legal guardians of children 0 to 6 reported demographic information; work, school, or childcare outside the home; infection prevention behaviors; and health history, including information related to COVID-19 vaccination and symptoms consistent with COVID-19. Participants who worked at an ILO were also asked more detailed questions about livestock production and processing activities. Study questionnaires were developed in collaboration with REACH organizers. REACH interviewers included those fluent in English and Spanish, and participants could respond in either language. REACH and JHSPH interviewers recorded participant responses in REDCap, a secure web application for managing online surveys (50, 51).

During the questionnaire, training, and sampling call, REACH or JHSPH interviewers instructed all enrolled participants on how to collect saliva samples and stayed on the call as participants collected samples to answer any questions and ascertain if participants followed procedures. All participants provided two self-collected saliva samples: an oral fluid saliva sample and a passive drool saliva sample. For the oral fluid sample, participants brushed the Oracol+ 2.0 saliva collection device (Malvern Medical Developments, Worcester, UK) along their gums for 1 to 2 minutes. Participants were instructed to store their samples in a refrigerator until pickup by a REACH courier or direct shipping to JHSPH. Because CDC vaccination recommendations changed during the course of our study, we added questions about this topic midstudy and recontacted participants.

### Multiplex SARS-CoV-2 IgG assay for oral fluid.

Oral fluid samples were separated from device sponges by centrifugation and tested for SARS-CoV-2 nucleocapsid (N), receptor-binding domain (RBD), and spike (S) IgG, using a multiplex bead-based immunoassay based on Luminex technology, which has been described previously 19, 20, 52. Briefly, the multiplex assay included SARS-CoV-2 N, RBD, and S antigens coupled to magnetic microparticles and a background control bead coated with bovine serum albumin (BSA). Saliva samples were centrifuged for 5 min at 10,000 × *g*, and 10 μL supernatant was added to a 96-well assay plate containing 40 μL bead mix (1,000 beads per bead set) in assay buffer (phosphate buffered saline with 0.045% Tween, 0.1% BSA, and 0.05% sodium azide). After incubation to allow for binding of SARS-CoV-2-specific IgG present in saliva samples, beads were washed, and fluorophore-labeled anti-human IgG was added to the plate. After a second incubation to allow for binding of labeled anti-IgG to salivary IgG on the beads, the plate was washed again, and the median fluorescent intensity (MFI) was read on a Luminex MagPix instrument.

To determine optimum performance cutoffs for infection-induced IgG, we used 1,320 saliva samples from individuals without known prior exposure to the SARS-CoV-2 virus or vaccine (presumed negatives) and 325 saliva samples collected >14 days after symptom onset of a molecularly confirmed SARS-CoV-2 infection (infection-induced positives). To determine optimum performance cutoffs for infection- and/or vaccination-induced IgG, we used 1,002 saliva samples from individuals without known prior exposure to the SARS-CoV-2 virus or vaccine (presumed negatives) and 492 saliva samples collected >14 days after symptom onset of a molecularly confirmed SARS-CoV-2 infection and/or >14 days after completing the primary COVID-19 vaccination series (infection- and/or vaccination-induced positives). We first subtracted the BSA signal from all SARS-CoV-2 signals. The best algorithm for infection-induced IgG relied on N (catalog [cat.] no. Z03480, Genscript, New Jersey, United States) and RBD (cat. no. 40592-V08H, Sino Biological, Beijing, China) (sensitivity, 97.6%; specificity, 99.4%), and the best algorithm for infection- and/or vaccination-induced IgG response relied on RBD (cat. no. 40592-V08H, Sino Biological) (sensitivity, 99.4%; specificity, 99.3%).

### Data from other southern United States cohorts.

Seropositivity data from the COVID-19 Prevention in Emory Healthcare Personnel (COPE) Study cohort were obtained through correspondence (M. H. Collins and C. D. Heaney, correspondence). Participants were health care providers recruited from 4 university-affiliated hospitals and clinics in Atlanta, Georgia, and saliva samples for serology were collected at enrollment, at 3 months, at 6 months, and at 9 months. Oral fluid saliva samples were tested with the multiplex assay described above as in our North Carolina study population (19, 20, 28, 29). Monthly seropositivity and N seropositivity data from the Cabarrus County COVID-19 Prevalence and Immunity (C3PI) study were also obtained through correspondence (L. K. Newby and D. Wixted, correspondence). Participants were selected from a larger ongoing cohort study through a weighted, randomized scheme to approximate the sex, age, and race/ethnicity of Cabarrus County, North Carolina. Blood samples for serology were collected monthly, and serology testing was performed with the Abbott Alinity IgG N protein antibody assay (specificity, 99.9%; sensitivity, 100%) (30). Nationwide and North Carolina infection-induced antibody seroprevalence estimates from CDC commercial laboratory surveys were downloaded from https://covid.cdc.gov/covid-data-tracker/#national-lab (35).

### Statistical analysis.

We first compared the distribution of demographic characteristics and potential risk factors for SARS-CoV-2 IgG among the ILO, ILON, and metro household groups. Using participants’ reported dates of receiving a first dose, receiving a second dose (or first dose for the Janssen [Johnson & Johnson] vaccine), and receiving a booster dose of the COVID-19 vaccine, we plotted the time to each vaccination event by group and tested for the difference in time to each vaccination event by group using the 3-group log-rank test implemented in the survdiff function (survival package) in R. Next, we calculated the crude prevalence of salivary SARS-CoV-2 IgG outcomes in each household group (ILO, ILON, and metro) and the crude prevalence of infection-induced IgG across levels of participant characteristics. For SARS-CoV-2 IgG and self-reported COVID-19 outcomes, we used generalized estimating equation (GEE) log-binomial regression models clustered by household to calculate crude prevalence ratios (PRs) and 95% confidence intervals (CIs) comparing outcome prevalence among the ILO versus the ILON, metro, and combined ILON and metro groups. We also used GEE log-binomial regression models clustered by household to calculate crude prevalence ratios of infection-induced IgG by participant characteristics, with the category with the greatest number of participants as the reference group. To compare infection-induced IgG prevalence in our cohort to other southern United States cohorts, we used log-binomial regression models to calculate crude prevalence ratios between the ILO group versus the ILON, metro, and other southern United States cohort groups with enrollment dates overlapping at least 1 day of our enrollment date range. All statistical analyses were completed in R 2022.02.0 (53).
