# Supplementary material for: SARS-CoV-2 Antibody Prevalence among Industrial Livestock Operation Workers and Nearby Community Residents, North Carolina, 2021 to 2022
Source: mSphere. 2023 Jan 19;8(1):e00522-22. doi: 10.1128/msphere.00522-22 (PMC9942583; doi:10.1128/msphere.00522-22)
Supplement: TABLE S1 [file msphere.00522-22-s0001.docx]

| **Characteristic** | | **ILO (n=90)** | **ILON (n=97)** | **Metro (n=92)** | **REACH study population (n=279)** | **North Carolina*^a^*** | **United States*^a^*** |
| --- | --- | --- | --- | --- | --- | --- | --- |
| Completed initial COVID-19 vaccination protocol, n (%) | |  |  |  |  |  |  |
|  | By December 29, 2021 (Q1) | 6/19 (31.6) | 18/35 (51.4) | 8/16 (50) | 32/70 (45.7) | (56.7) | (61.9) |
|  | By January 10, 2022 (Q2) | 12/33 (36.4) | 40/70 (57.1) | 19/37 (51.3) | 71/140 (50.7) | (57.3) | (62.6) |
|  | By March 3, 2022 (Q3) | 32/60 (53.3) | 44/96 (45.8) | 28/54 (51.9) | 105/210 (50) | (59.4) | (65) |
|  | By July 18, 2022 (Q4) | 47 (51.6) | 47 (48.5) | 51 (55.4) | 145 (52) | (62.9) | (67.2) |
|  |  |  |  |  |  |  |  |
| Received at least one booster dose, n (%) | |  |  |  |  |  |  |
|  | By December 29, 2021 (Q1) | 1/19 (5.3) | 1/35 (2.9) | 0 (0) | 2/70 (2.9) | (11.3) | (20.5) |
|  | By January 10, 2022 (Q2) | 3/33 (9.1) | 9/70 (12.9) | 5/37 (13.5) | 17/140 (12.1) | (12.5) | (22.8) |
|  | By March 3, 2022 (Q3) | 6/60 (10) | 10/96 (10.4) | 10/54 (18.5) | 26/210 (12.4) | (15) | (28.5) |
|  | By July 18, 2022 (Q4) | 12 (13.2) | 10 (10.3) | 19 (20.7) | 41 (14.7) | (17.7) | (32.3) |
|  |  |  |  |  |  |  |  |
| Answered follow-up call, n (%) | | 73 (81.1) | 88 (90.7) | 82 (89.1) | 243 (87.1) | - | - |
|  | |  |  |  |  |  |  |
| Follow-up or interview (if did not answer follow-up) call dates, median (IQR) | | 2/10/2022 (12/29/2021-3/29/2022) | 1/3/2022 (11/3/2021-1/11/2022) | 1/11/2022 (1/03/2022-4/11/2022) | 1/10/2022 (12/29/2021-3/2/2022) | - | - |
