# Supplementary figures and images for: SARS-CoV-2 Antibody Prevalence among Industrial Livestock Operation Workers and Nearby Community Residents, North Carolina, 2021 to 2022
Source: mSphere. 2023 Jan 19;8(1):e00522-22. doi: 10.1128/msphere.00522-22 (PMC9942583; doi:10.1128/msphere.00522-22)

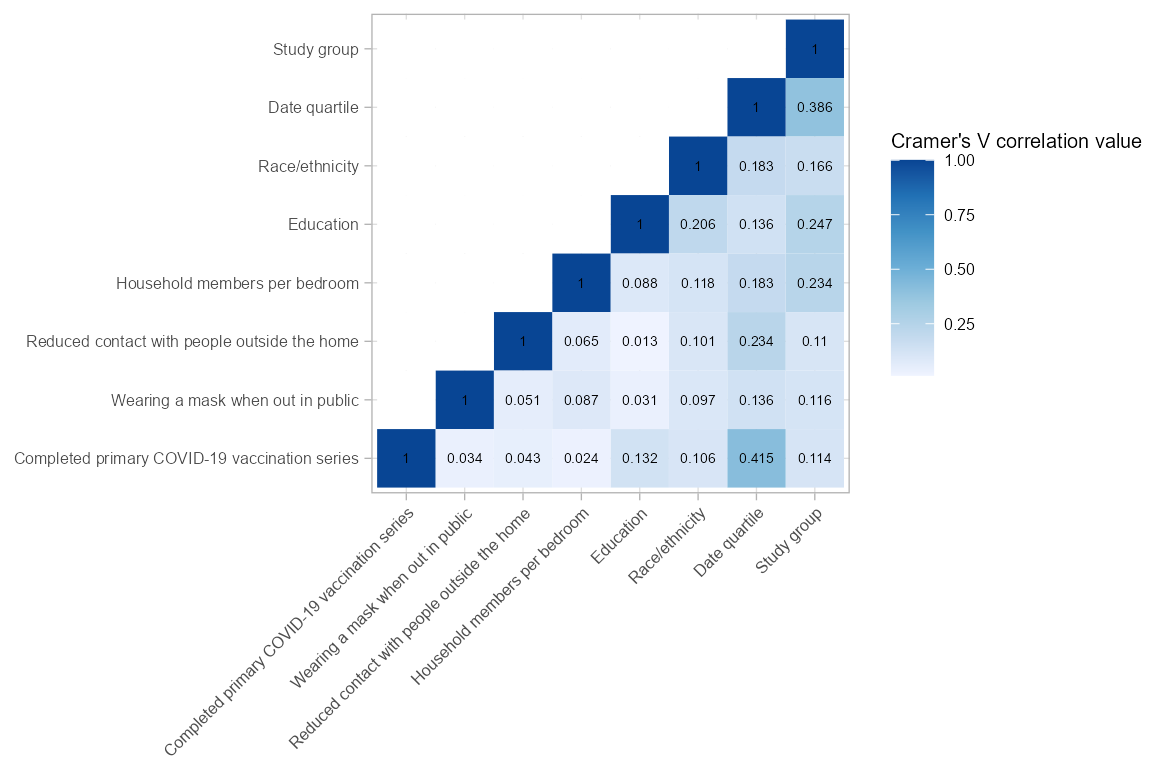

Supplement: FIG S1 [file msphere.00522-22-s0003.tif]

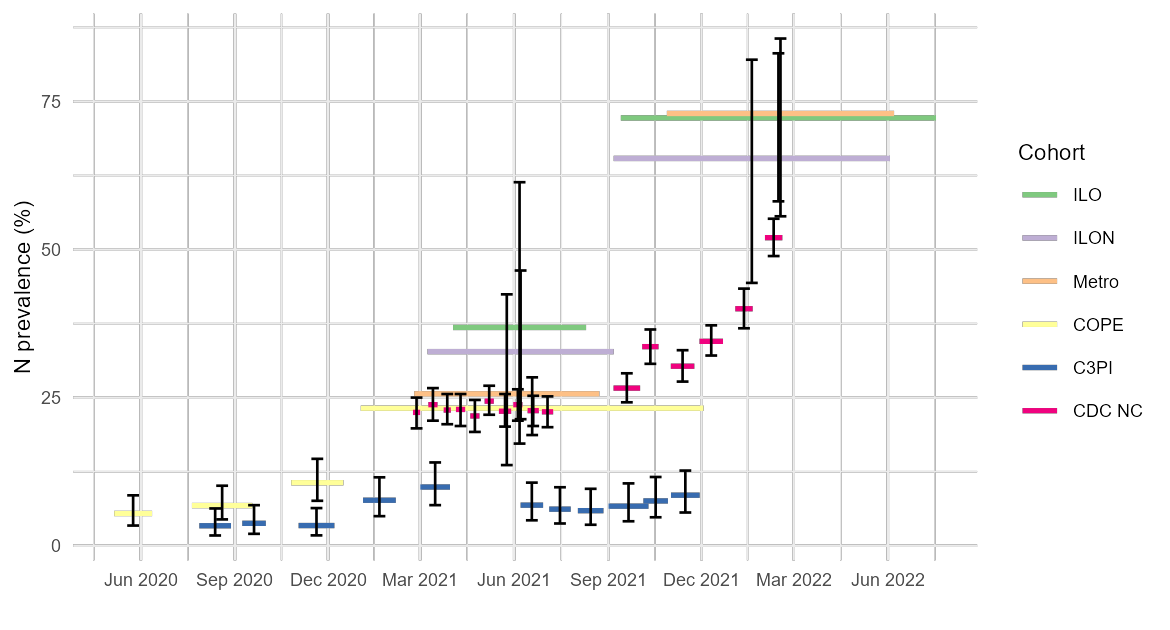

Supplement: FIG S2 [file msphere.00522-22-s0004.tif]
