# Supplementary material for: SARS-CoV-2 Antibody Prevalence among Industrial Livestock Operation Workers and Nearby Community Residents, North Carolina, 2021 to 2022
Source: mSphere. 2023 Jan 19;8(1):e00522-22. doi: 10.1128/msphere.00522-22 (PMC9942583; doi:10.1128/msphere.00522-22)
Supplement: TABLE S2 [file msphere.00522-22-s0002.docx]

| **Characteristic, n (%)** | | **ILO n=90** | | **ILON n=97** | | **Metro n=92** | | **p-value*^a^*** |
| --- | --- | --- | --- | --- | --- | --- | --- | --- |
|  |  | **(80 households)** | | **(80 households)** | | **(80 households)** | |  |
| **Infection prevention behaviors** | |  |  |  |  |  |  |  |
| Reduced physical contact with people outside your home | |  |  |  |  |  |  | 0.16 |
|  | Yes, all household members | 65 | (71.4) | 81 | (83.5) | 69 | (75) |  |
|  | Yes, some but not all household members | 10 | (11) | 4 | (4.1) | 6 | (6.5) |  |
|  | No | 9 | (9.9) | 4 | (4.1) | 7 | (7.6) |  |
|  | No response | 7 | (7.7) | 8 | (8.2) | 10 | (10.9) |  |
| Avoiding or cancelling travel or vacation plans | |  |  |  |  |  |  | 0.05 |
|  | Yes | 72 | (79.1) | 75 | (77.3) | 56 | (60.9) |  |
|  | No | 19 | (20.9) | 21 | (21.6) | 33 | (35.9) |  |
|  | No response | 0 |  | 1 | (1) | 3 | (3.3) |  |
| Wearing a mask when out in public | |  |  |  |  |  |  | 0.16 |
|  | Yes | 86 | (94.5) | 93 | (95.9) | 83 | (90.2) |  |
|  | No | 5 | (5.5) | 3 | (3.1) | 9 | (9.8) |  |
|  | No response | 0 | (0) | 1 | (1) | 0 | (0) |  |
| Washing hands/using hand sanitizer more frequently | |  |  |  |  |  |  | 0.62 |
|  | Yes | 88 | (96.7) | 90 | (92.8) | 86 | (93.5) |  |
|  | No | 3 | (3.3) | 5 | (5.2) | 5 | (5.4) |  |
|  | No response | 0 | (0) | 2 | (2.1) | 1 | (1.1) |  |
| At times (last 2 w) you interacted with people inside … | |  |  |  |  |  |  |  |
| How often did you maintain a 6 ft distance from others? | |  |  |  |  |  |  | 0.41 |
|  | Always | 67 | (73.6) | 78 | (80.4) | 68 | (73.9) |  |
|  | Sometimes | 24 | (26.4) | 17 | (17.5) | 21 | (22.8) |  |
|  | Never | 0 | (0) | 1 | (1) | 2 | (2.2) |  |
|  | No response | 0 | (0) | 1 | (1) | 1 | (1.1) |  |
| How often did you wear a mask? | |  |  |  |  |  |  | 0.06 |
|  | Always | 74 | (81.3) | 82 | (84.5) | 66 | (71.7) |  |
|  | Sometimes | 17 | (18.7) | 13 | (13.4) | 22 | (23.9) |  |
|  | Never | 0 | (0) | 1 | (1) | 4 | (4.3) |  |
|  | No response | 0 | (0) | 1 | (1) | 0 | (0) |  |
| **Health history** | |  |  |  |  |  |  |  |
| Do you have any of the following chronic conditions? | |  |  |  |  |  |  |  |
|  | Hypertension | 25 | (27.5) | 28 | (28.9) | 22 | (23.9) | 0.77 |
|  | Diabetes | 15 | (16.5) | 17 | (17.5) | 10 | (10.9) | 0.43 |
|  | Asthma | 9 | (9.9) | 5 | (5.2) | 13 | (14.1) | 0.10 |
|  | Depression | 2 | (2.2) | 6 | (6.2) | 12 | (13) | 0.03 |
|  | Cardiovascular disease | 2 | (2.2) | 6 | (6.2) | 5 | (5.4) | 0.38 |
|  | Cancer diagnosis/treatment (past 12 mo) | 4 | (4.4) | 3 | (3.1) | 1 | (1.1) | 0.41 |
|  | Chronic kidney disease | 1 | (1.1) | 4 | (4.1) | 3 | 3.3 | 0.44 |
|  | Autoimmune disease | 1 | (1.1) | 2 | (2.1) | 2 | 2.2 | 0.83 |
|  | Chronic obstructive pulmonary disease (COPD) | 2 | (2.2) | 1 | (1) | 1 | (1.1) | 0.76 |
|  | Immunocompromised condition | 2 | (2.2) | 0 | (0) | 1 | (1.1) | 0.35 |
|  | Other chronic lung disease | 2 | (2.2) | 1 | (1) | 0 | (0) | 0.36 |
|  | Sickle cell anemia | 1 | (1.1) | 0 | (0) | 1 | (1.1) | 0.59 |
| Do you have seasonal allergies? | | 42 | (46.2) | 49 | (50.5) | 57 | (62) | 0.04 |
| Have you gotten or plan to get a flu shot this year? | | 36 | (39.6) | 48 | (49.5) | 44 | (47.8) | 0.30 |
| Fever with a cough at the same time or fever with a sore throat at the same time (past yr)?*^b^* | | 19 | (20.9) | 15 | (15.5) | 17 | (18.5) | 0.33 |
| Since February 1, 2020, have you thought you had COVID-19? | | 22 | (24.2) | 18 | (18.6) | 18 | (19.6) | 0.28 |
