# Supplementary material for: SARS-CoV-2 Antibody Prevalence among Industrial Livestock Operation Workers and Nearby Community Residents, North Carolina, 2021 to 2022
Source: mSphere. 2023 Jan 19;8(1):e00522-22. doi: 10.1128/msphere.00522-22 (PMC9942583; doi:10.1128/msphere.00522-22)
Supplement: TABLE S3 [file msphere.00522-22-s0005.docx]

| **Characteristic, n (%)** | | **ILO (n=90)** | **ILON (n=97)** | **Metro (n=92)** | **REACH study population (n=279)** | **COPE (n=306)** | **C3PI (n=300)** |
| --- | --- | --- | --- | --- | --- | --- | --- |
| SARS-CoV-2 infection-induced IgG*^a^* | | 46/73 (63) | 36/84 (43) | 37/76 (49) | 119/233 (51.1) | 71/306 (23.2) | 23/270 (8.5) |
| Sampling date, median (range) | | 2/9/2022 (4/2/2021-7/18/2022) | 6/6/2021 (3/8/2021-6/3/2022) | 8/18/2021 (2/23/2021-6/7/2022) | 9/6/2021 (2/23/2021-7/18/2021) | 2/25/2021 (1/2/2021-12/3/2021) | — |
|  |  |  |  |  |  |  | Latest 11/2021 |
| Age in years, median (range) | | 41.5 (13-67) | 50 (5-83) | 37 (9-74) | 41 (5-83) | 37 (22-71) | — |
|  |  |  |  |  |  |  | Median 56 (IQR 47, 67) |
| Gender | |  |  |  |  |  |  |
|  | Female | 47 (52) | 54 (56) | 67 (73) | 139 (59.7) | 233 (76.1) | 183 (61.0) |
|  | Male | 43 (48) | 42 (43) | 25 (27) | 94 (40.3) | 73 (23.9) | 117 (39.0) |
| Race/ethnicity | |  |  |  |  |  |  |
|  | Black/African American | 79 (88) | 83 (86) | 74 (80) | 236 (84.5) | 41 (13.4) | 51 (17.0) |
|  | Hispanic/Latino | 8 (9) | 11 (11.3) | 3 (3.3) | 22 (7.9) | 13 (4.2) | 33 (11.0) |
|  | White/Caucasian | 1 (1.1) | 1 (1) | 8 (8.7) | 10 (3.6) | 217 (71.0) | 230 (76.7) |
|  | Both Black and White | 0 (0) | 1 (1) | 2 (2.2) | 3 (1.1) | 2(0.01) | — |
|  | Asian-American | 0 (0) | 0 (0) | 1 (1) | 1 (0.004) | 29 (9.5) | 0 (0) |
|  | Other or no response | 2 (2.2) | 1 (1) | 3 (3.3) | 6 (2.2) | 17 (5.6) | — |
| Education | |  |  |  |  |  |  |
|  | ≤High school/GED | 65 (71) | 53 (55) | 37 (40) | 155 (55.6) | — | 19 (6.3) |
|  | Post-high school | 26 (29) | 42 (43) | 53 (58) | 121 (43.3) | — | 180 (93.3) |
|  | No response | 0 (0) | 2 (2.2) | 2 (2.2) | 4 (1.4) | — | 1 (0.3) |
| Household members, mean (SD) | | 2.7 (1.4) | 2.1 (1.3) | 2.2 (1.2) | 2.3 (1.3) | — | Median 2 (IQR 2, 4) |
| Health insurance*^b^* | |  |  |  |  |  |  |
|  | Company health insurance | 30 (33) | 33 (34) | 56 (61) | 119 (42.7) | — | 169 (56.3) |
|  | Public health insurance | 34 (38) | 35 (36) | 17 (19) | 86 (30.8) | — | 94 (31.3) |
|  | Private health insurance | 15 (17) | 16 (17) | 10 (11) | 41 (14.7) | — | 12 (4.2) |
|  | No health insurance | 12 (13) | 15 (16) | 8 (9) | 35 (12.5) | — | 14 (4.7) |
|  | Other or no response | 2 (2) | 0 (0) | 1 (1.1) | 3 (1.1) | — | 25 (8.3) |
| Completed COVID-19 primary vaccination series | | 47 (51.6) | 47 (48.5) | 51 (55.4) | 145 (52) | 249 (81.4) | — |
