# Supplementary material for: SARS-CoV-2 Antibody Prevalence among Industrial Livestock Operation Workers and Nearby Community Residents, North Carolina, 2021 to 2022
Source: mSphere. 2023 Jan 19;8(1):e00522-22. doi: 10.1128/msphere.00522-22 (PMC9942583; doi:10.1128/msphere.00522-22)
Supplement: TABLE S4 [file msphere.00522-22-s0006.docx]

| **Vaccination status at sampling date** | | **ILO (n=73)** | **ILON (n=84)** | **Metro (n=76)** | **REACH study population (n=233)** | **COPE (n=305)** |
| --- | --- | --- | --- | --- | --- | --- |
| Completed primary COVID-19 vaccination series, and at least 2 weeks post-completion, no. pos./total (%) | | 23/32 (72) | 19/29 (66) | 19/35 (54) | 61/96 (64) | 38/214 (18) |
|  | |  |  |  |  |  |
| Not at least 2 weeks post-primary COVID-19 vaccination series completion, no. pos./total (%) | | 23/41 (56) | 17/55 (31) | 18/41 (44) | 58/137 (42) | 33/91 (36) |
|  | No record of any COVID-19 vaccination, no. pos./total (%)^a^ | 19/32 (59) | 13/36 (36) | 13/28 (46) | 45/96 (47) | 23/58 (40) |
|  | Received at least first dose of COVID-19 vaccination series, no. pos./total (%)^a^ | 25/38 (66) | 20/36 (56) | 20/42 (48) | 65/116 (56) | 43/231 (19) |
|  | Completed primary COVID-19 vaccination series, no. pos./total (%)^a^ | 23/33 (70) | 19/30 (63) | 19/37 (51) | 61/100 (61) | 41/225 (18) |
